# Development of a Unified Specimen for Adhesive Characterization—Part 2: Experimental Study on the Mode I (mDCB) and II (ELS) Fracture Components

**DOI:** 10.3390/ma17051049

**Published:** 2024-02-24

**Authors:** Daniel S. Correia, Inês D. Costa, Eduardo A. S. Marques, Ricardo J. C. Carbas, Lucas F. M. da Silva

**Affiliations:** 1 Institute of Science and Innovation in Mechanical and Industrial Engineering (INEGI), University of Porto, Rua Dr. Roberto Frias 400, 4200-465 Porto, Portugal; dcorreia@inegi.up.pt (D.S.C.);; 2 Department of Mechanical Engineering, Faculty of Engineering (FEUP), University of Porto, Rua Dr. Roberto Frias 400, 4200-465 Porto, Portugal; emarques@fe.up.pt (E.A.S.M.); lucas@fe.up.pt (L.F.M.d.S.)

**Keywords:** structural adhesives, adhesive characterization, fracture toughness, fracture process zone, unified specimen

## Abstract

Adhesive bonding has been increasingly employed in multiple industrial applications. This has led to a large industrial demand for faster, simpler, and cheaper characterization methods that allow engineers to predict the mechanical behavior of an adhesive with numerical models. Currently, these characterization processes feature a wide variety of distinct standards, specimen configurations, and testing procedures and require deep knowhow of complex data-reduction schemes. By suggesting the creation of a new and integrated experimental tool for adhesive characterization, it becomes possible to address this problem in a faster and unified manner. In this work, following a previous numerical study, the mode I and II components of fracture-toughness characterization were validated experimentally in two different configurations, Balanced and Unbalanced. For mode I, it was demonstrated that both configurations presented similar numerical and experimental R-curves. The relative error against standard tests was lower than ±5% for the Balanced specimen; the Unbalanced system showed higher variations, which were predicted by the numerical results. Under mode II, the Balanced specimen displayed plastic deformation due to high deflections. On the contrary, the Unbalanced specimen did not show this effect and presented a relative error of approximately ±2%. Nonetheless, it was proven that this approach to obtain such data by using a single unified specimen is still feasible but needs further development to obtain with similar precision of standard tests. In the end, a conceptual change is proposed to solve the current mode II issues.

## 1. Introduction

The dissemination of the adhesive bonding processes as a complement to or even replacement for conventional mechanical joining techniques is taking place at a rapid pace in several different industries. This change in paradigm requires the support of new designs and construction concepts. As such, to ensure the accurate optimization of the performance of complex bonded connection, numerical simulation must be performed.

Great strides have been made to comprehend the science behind adhesive bonding and the mechanical properties of adhesives, mostly since these properties, strength and toughness, are required to define the cohesive laws necessary to run the previously mentioned simulations. These assist structural engineers in adhesive selection and structural-performance optimization during the design phase.

Apart from stiffness and strength assessments, which have been widely studied, toughness has been only more recently accessed through fracture mechanics approaches [1]. These were developed to predict the failure of adhesive joints.

Experimentally, fracture mechanics tests mainly intend to measure a parameter that is, ideally, independent of the geometry of the cracked body or the thickness of the adhesive layer [2]. As such, this “material parameter” is used for characterizing the toughness of materials, including in this case both interfaces and adhesives. One such parameter is the critical energy release rate, *G*_C_, also called fracture toughness, which can be defined in three modes: mode I (tensile opening mode), mode II (in-plane shearing mode), and mode III (out-of-plane shearing mode) [2].

However, recent studies by Akhavan-Safar et al. [3], for mode I specimens, and Delzendehrooy et al. [4], for mode II loading, have shown otherwise. *G*_C_ might not be fully independent of the geometry and mechanical characteristics of the specimen. By analyzing the results of numerous studies, with adhesives being tested with different adherend geometries and materials, several dependencies were found, placing doubts on the fact that the *G*_C_ can be defined as a material property. Additionally, Sarrado et al. [5] proved that besides the geometry the data reduction scheme type may also influence the results. There are two main types, the simpler linear elastic fracture mechanics (LEFMs), and the more versatile non-linear fracture mechanics (NLFM).

Nonetheless, as part of the prevailing procedure, the concept of fracture energy of an adhesive as a material property remains, at least whenever a fully cohesive failure of the adhesive occurs. A similar concept can also be defined along the adhesive/substrate interface if the joint presents interfacial failure, in this case, representing a property of the specific interface and not of the adhesive. These fracture-mechanics-based procedures have been developed for characterizing and predicting the failure of adhesive joints, including flexible laminates [1].

**Figure 1:**
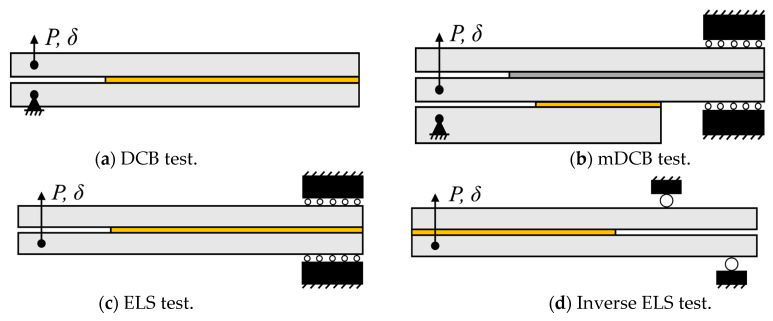
Mode I and II adhesive characterization specimens. Active/relevant adhesive layer is presented in yellow.

The double cantilever beam (DCB)—Figure 1a—introduced by Ripling and Mostovoy [6,7] in the 1960s, is the most commonly used mode I test specimen. Employing a simple design, resulting in low-cost adherends, it has become the standard for determining the critical strain energy release rate in mode I, that is, *G*_IC_ [8]. Such specimens are currently even more appealing since several crack-independent data reduction schemes have been developed [9,10]. This removes the previous need to directly monitor crack propagation [1].

Since the 1960s, researchers have studied in detail the DCB test, resulting in the development of the ASTM D3433 standard [11]. Published in 1973, it was later revised by Blackman et al. [12], resulting in the publication of BS 7991 [11] in 2001. The growing use of fiber-reinforced polymer composites has led, as of late, to the publication of a new international standard, known as ISO 25217 [11].

Currently, only composite materials have standardized tests for pure mode II fracture toughness (*G*_IIC_) characterization [8]. One of the most commonly used methods is the end-loaded split (ELS) test—Figure 1c—which provides mode II loading through flexion, resulting in pure shear in the specimen’s middle plane [1,13]. When compared against other testing procedures like the end-notched flexion (ENF), four-point ENF (4ENF), or full mode II mixed moment bending (MMB), the ELS has proved to be the better candidate [14]. This mode II test does, however, still present some limitations related to friction, crack propagation stability, and proper fracture process zone (FPZ) development.

Due to the large ductility of the adhesive layers in adhesively bonded joints, the appearance of larger FPZs has been reported mostly under shear loading. And since the adhesive layer thickness is usually quite small, the common crack/FPZ monitoring techniques used in mode I present enormous challenges under mode II loading. As such, crack-independent methods, like the J-integral [15,16], and the effective crack length, *a*_eq_, [9,17,18,19,20], have been developed to address this issue.

This research has provided encouraging results, which, together with a simple manufacturing process like that of DCB specimens, make it an attractive test. One such benefit is the advantage of having stable crack propagation under displacement control in composite joints if the initial crack (*a*_0_) to span length (*L*) ratio is higher than 0.55 [8,19,21,22,23].

Regarding the application of the ELS test in adhesive characterization, according to recent studies, a few requirements must be satisfied in order to ensure stable crack propagation during an ELS test. First, it is necessary to guarantee the formation of the entire FPZ by achieving steady-state crack propagation and, consequently, the plateau of the resistance curve. Secondly, it is necessary to guarantee the stability of the test under displacement control. The third requirement is to prevent significant deflections of the specimen. And the fourth requirement is to prevent adherend failure while testing [24]. To meet these conditions, the ELS specimen’s geometry can be changed, and the span length (*L*_ELS_), initial crack length (*a*_0ELS_), and, if necessary, the specimen’s beam height (*h*_ELS_) must be altered to achieve these conditions [24,25]. However, these criteria can be difficult to meet from a practical standpoint.

As such, alternative tests must be developed since the ELS tests still present excessive friction between the unbonded beams and instabilities associated with the crack propagating in the direction of the highest flexural moment. A novel method was devised by Budzik and Jummel [26,27], the inverse ELS (I-ELS) test—Figure 1d—which is inherently a stable concept. This is due to the crack propagating in the direction of the highest flexural moment, improving on the fact that the ELS test was simply conditionally stable.

Fracture testing results, being highly dependent on data-reduction methods to extract the intended properties from the *P*-*δ* curves, can be extremely complex to analyze. Two main types of formulations can be devised, based on either LEFM [9,28] or NLFM, like the J-Integral [29,30].

Most classical LEFM data-reduction schemes [9,28] are based on the Irwin–Kies equation but usually need crack monitoring, which can be difficult to achieve, especially under mode II loading. Some of these classical methods derive from the simple beam theory (SBT) and include the compliance calibration method (CCM), the direct beam theory (DBT), the enhanced simple beam theory (ESBT) [31], and the corrected beam theory (CBT). Each one attempts to improve on the previous one to account for the deviations from the simple assumptions of SBT, from experimental compliance calibration in CCM, to correction factors, like the ones proposed by Hashemi et al. [32] that take into account the end block stiffening and large deflections. This later resulted in the correction factor ∆ to account for crack tip rotation and deflection, proposed in 1992 by Wang and Williams, for both mode I [33] and mode II [34] specimens. Nonetheless, with all these innovations, there is still the need to measure the real crack propagation, which is experimentally troublesome in mode II loading. When extensive damage occurs, such as microcracking or fiber bridging, this also becomes a problem [35].

In recent years, approaches based on the concept of the equivalent crack length started to emerge because they were independent of crack monitoring. The compliance-based beam method (CBBM) [9,18] is one of these approaches. It considers three main aspects, the Timoshenko beam theory, the specimen’s compliance, and the equivalent crack length concept. Also considering the FPZ effects, this method became especially important for ductile adhesives and mode II loading. Since its initial appearance for the DCB specimen, several new formulations have been devised, under mode I, for DCB [9], tapered DCB [36], and modified DCB [37], and under mode II, for the end-notched flexure (ENF) [18] and ELS [18] tests, and also several mixed-mode specimens [11,13].

At the moment, there are multiple specimens developed to characterize the mechanical behavior of the adhesives to be studied, under pure tensile and shear loading or fracture under mode I or II loading conditions. However, no test simultaneously combines more than one pure loading condition. Conclusively, to obtain the mechanical properties necessary to define the cohesive laws of an adhesive, four specimens, four testing procedures, and four data-reduction schemes are necessary, one for each loading condition. As such, adhesive characterization becomes a complex, time-consuming, and costly procedure for non-specialized personnel. Other than having to cure and perform four batches of tests separately with their specific apparatuses, it is necessary to have proper knowledge on how to manufacture, test, and treat the obtained data. Therefore, to a company, the competitiveness associated with not having to hire third parties to characterize the materials under inquiry becomes highly appealing.

For this purpose, a novel experimental tool [38] is being developed at INEGI (Porto, Portugal). This tool consists of a unified specimen with four adhesive layers; a test apparatus, which sequentially tests all loading conditions in a single vertical movement; and a reduction code, which treats the raw data and gives the wanted properties. The full concept combines a butt joint, for tensile strength; the modified thick adherend shear test (mTAST), for shear strength; the modified DCB (mDCB), for mode I fracture toughness; and the ELS, for mode II fracture toughness.

The present work intends to experimentally validate fracture components of this concept, which were numerically studied previously by Correia et al. [37]; see Figure 1b. As previously shown by Faria et al. [38] and Correia et al. [37], the combined specimen is able to isolate the propagation of each mode without interference. This enables each test to be analyzed independently, since for mode I the whole specimen (Figure 1b) is being used. And for mode II, only the two upper beams are under consideration, since at this point, the mDCB’s lower beam completely debonded from the combined specimen (turning Figure 1b into Figure 1c).

This work was performed using the same two structural epoxy adhesives used in the previously mentioned study, allowing us to validate the experimental capabilities of this specimen against the numerical data obtained then. Considering the design recommendations presented by Correia et al. [37], two conditions were tested experimentally. They were defined as Balanced and Unbalanced specimen configurations, both with the optimal crack relations reported in the paper. The reasoning behind the names chosen is better detailed in Section 2.2.

As an outcome, it was proven, experimentally, that the mDCB specimen can properly characterize an adhesive when using the recommended dimensions. The ELS specimen presented issues related to crack propagation stability and plastic yielding of the substrate, resulting in a proposal for a conceptual change by introducing the I-ELS as a substitute for the ELS test.

## 2. Experimental Details

This study was developed as a continuation of a previous numerical investigation by Correia et al. [37]. A combined test intended to obtain the mode I and mode II energy release rate, merging two adhesive characterization concepts.

In this section, the experimental details and procedures used to manufacture, test, and analyze a unified specimen are described; see Figure 1b, first focusing on common aspects and then advancing to specific considerations relative to the study of the mDCB test and the ELS test.

Since these configurations are sequentially tested, starting with the mode I component and then transitioning to the mode II test, they can be looked at separately and in this order.

To simplify the equations in terms of variables, parameters associated with the mDCB adhesive layer or mDCB lower arm are referred to as “I”, and similarly, for the ELS they are referred to as “II”.

### 2.1. Materials

In this work, two epoxy adhesives were considered, both tested with high-strength steel adherends. Their properties and other particularities are described in the next sub-sections.

#### 2.1.1. Adhesive

Two different adhesives were studied to experimentally validate on a wider spectrum the new tool concept for the combined test of mode I and mode II fracture characterization.

Both have high strength and stiffness [37,39], since their working temperature is below the glass transition temperature (*T*_g_), at 80 °C and 130 °C, respectively. Their curing mechanism is heating with amine-based hardeners.

The first is brittle in nature and, as such, is referred to as “brittle adhesive”. It is a structural two-component (2K), room-temperature curing paste adhesive that can have an accelerated curing at higher temperatures. This adhesive’s fracture properties were obtained by Correia et al. [37] to proceed with the numerical study that preceded this work. The curing cycle used was 100 °C for 5 min, as recommended by the manufacturer, without considering heating and cooling ramps.

The second adhesive of a tougher nature has been fully characterized by Correia et al. [39] in a non-related work. In this paper, it was referred to as a “tough adhesive” due to its higher toughness in comparison to the previously used adhesive. It is a one-part (1K) structural epoxy resin that is heat-cured. The curing cycle used was 150 °C for 40 min, as recommended by the manufacturer, without considering heating and cooling ramps.

The resins were cured using the same cycles used in previous works, where they were standardly characterized, to make the results comparable.

The relevant mechanical properties of each adhesive are compiled in Table 1, according to each respective work where they were characterized.

The reference values of *G*_IC_ and *G*_IIC_ were obtained through DCB and ENF tests, respectively.

#### 2.1.2. Substrates

The substrates used in this work were manufactured from two grades of high strength steel from Ramada Aços (Ovar, Portugal). An alloyed construction steel, DIN 40CrMnMo7, was used for the ticker 12.7 mm substrates. And a ledeburitic high carbon tool steel, DIN X155CrVMo12-1, was used for the thinner 9 mm substrates. Both were employed to avoid plastic deformation during the tests, as any yielding generates inaccuracies in the results obtained by the fracture toughness characterization tests. But each depends on the hardness needed for the substrate thicknesses involved.

Table 2 displays some of the mechanical properties of the substrates.

### 2.2. Unified Specimen—mDCB Plus ELS

**Figure 2:**
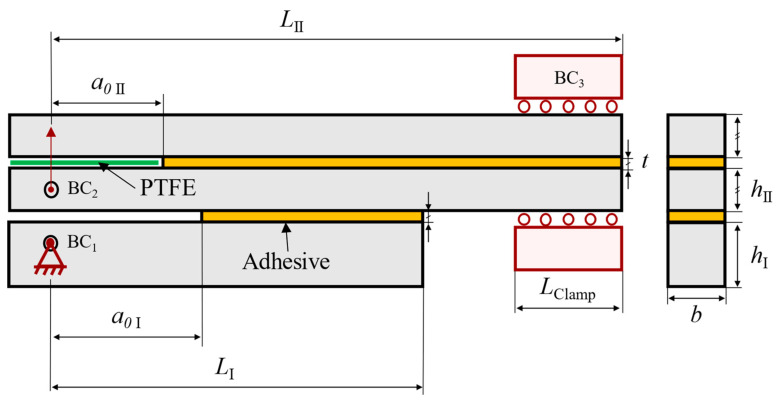
Schematic representation of the mDCB (“I”) and ELS (“II”) fracture specimen, with its associated dimensions, testing procedure, and respective boundary condition (BC).

Following the numerical study [37] performed prior to this work, the unified specimen was designed as described in Figure 2. Both the characteristic dimensions and boundary conditions are described in the scheme.

Experimentally, several factors could be enhanced in relation to the numerical study as a consequence of having a composed specimen—e.g., the FPZ, substrate yielding, interactions between both cracks, and others. As such, two conditions were analyzed against their numerical results, a Balanced and an Unbalanced configuration. The unified specimen configurations to be tested have the dimensions shown in Table 3.

It is important to note that the crack lengths used for the tough adhesive were not the ones recommended by the numerical study. Both values were increased by 20 mm, resulting in the crack lengths seen in Table 3. This change was performed to further improve ELS crack stability and reduce even more the possibility of plastic deformation in the ELS test. This was solely performed for the tough adhesive, as it is the more critical case in this regard.

As briefly mentioned in Section 1, two configurations were tested, named Balanced and Unbalanced. Their dimensional difference lay in parameter *h*_II_, as depicted in Table 3. The nomenclatures used are defined by the (*h*_I_, *h*_II_) relation depicted in Table 3 and mostly concern the behavior of the mDCB test. A Balanced configuration presents a numerical compliance ratio (*r*, see Section 2.3.1 and Equation (2)) of 0.5, which suggests equal rotation of each specimen arm. All other *h* configurations that present different ratios were therefore called Unbalanced. The Unbalanced configuration tested in this study presented a compliance ratio of 0.7.

#### Manufacturing

**Figure 3:**
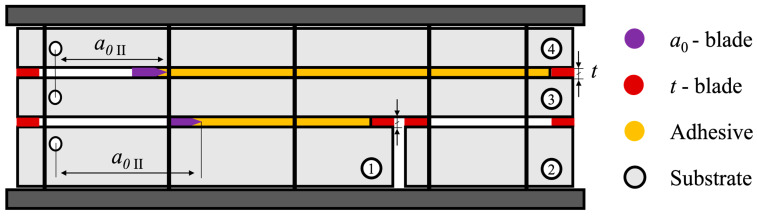
Schematic representation of the unified mode I and II fracture specimen in the manufacturing stage.

As mentioned above, the combined test merges a DCB with an ELS geometry, resulting in a modified DCB specimen. As such, these unified specimens were manufactured following the same procedures as DCB and ELS specimens. However, being a composed specimen, its manufacturing procedure followed a different staking sequence, as seen in Figure 3.

In accordance with the procedures detailed by Banea et al. [40], a proper steel mold was used, as well as the necessary pressure conditions. Prior to manufacturing, the steel mold and respective support pins were cleaned with high-grit sandpaper and degreased with acetone. Three layers of demolding agent (Loctite^®^ Frekote 770-NC, Henkel Adhesives, Düsseldorf, Germany) were applied to prevent adhesion.

The CNC-machined high-strength steel adherends (Section 2.1.2) were treated to increase their surface energy values. As such, the interfaces to be bonded were grit-blasted with alumina grit at 0.6 MPa of pressure and degreased with acetone, to improve adhesion and guarantee cohesive failure.

Two thin 0.1 mm-thick razor blades (“*a*_0_-blade” in Figure 3) were positioned in the middle of the bond line to promote the formation of a sharper initial crack, *a*_0_. Calibrated tapes (“*t*-blade” in Figure 3) were used to set an adhesive thickness (*t*) of 0.2 mm. All blades were coated with a demolding agent to prevent adhesion.

The assembly of the specimen was as detailed by the adherend numeration in Figure 3, applying the adhesive in each respective area between substrate staking. Substrate 2 was introduced to offer support and allow a proper pressure distribution, maintaining the correct alignment of the specimen and ensuring uniform adhesive thickness.

Following the assembly of the specimen, the adhesive was cured in a hot plate press at 2 MPa of pressure and following each curing condition described in Section 2.1.1, depending on the adhesive used.

After the curing cycle was completed, the specimens were removed from the mold, and the excess adhesive was removed using a manual milling machine, steel files, and sandpaper to create a smooth edge.

Additionally, the ELS adhesive layer was loaded under mode I prior to testing to remove the blade introduced in the manufacturing procedure, which would hinder crack propagation and to promote a stable crack propagation under future mode II testing conditions by creating a natural crack. The new initial crack length, *a*_0 II_, was then directly measured. The specimen was then ready to be assembled in the newly designed testing setup proposed for this kind of test.

### 2.3. Mechanical Characterization Test Procedures

**Figure 4:**
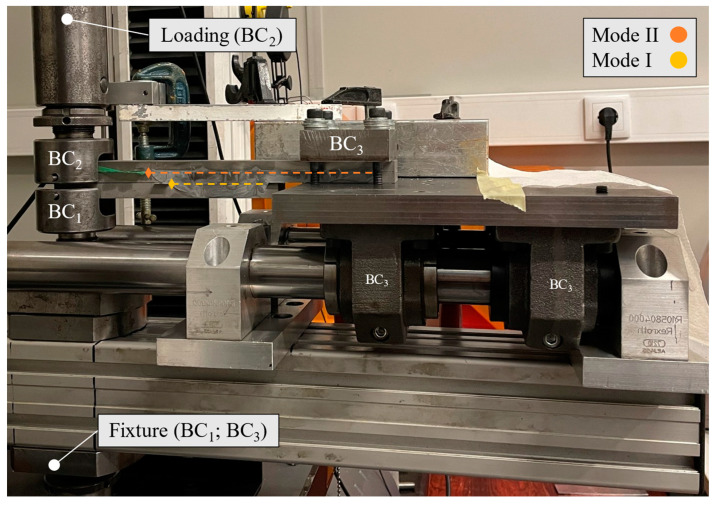
Experimental apparatus of the unified mode I and II fracture specimen. Each boundary condition is marked as seen in Figure 2. Dashed lines represent the adhesive layers.

All tests were performed using a universal testing machine, more specifically an INSTRON^®^ 3367 (Norwood, MA, USA) whose load cell is capable of measuring up to 30 kN with high precision, even at low loads. For both mode I and mode II loading, a quasi-static rate of 0.2 mm/min was used. The tests were performed in a prototype of the full apparatus, Figure 4, which was built to respect the boundary conditions depicted in Figure 2.

Boundary conditions 1 (BC_1_) and 2 (BC_2_) used simple U-shaped rods that were fixed to the base and moving shaft of the machine. Boundary condition 3 (BC_3_) was built using a stiff steel base mounted on top of four roller bearings, which were coupled to a guiding system. All this allows the necessary horizontal movement and ensures high flexural stiffness. To address the vertical clamping requirement, a steel block was placed on top of the clamping area of the specimen and then fixed to the steel base with four screws.

This setup was assembled in the universal testing machine, and the specimen was loaded through vertical displacement in an ascending movement, precisely replicating the required loading conditions of both the mDCB and ELS tests.

To attain the fracture toughness in each mode, the CBBM was employed to analyze the load–displacement curves obtained from the universal testing machine, resulting in the calculation of the respective R-curves.

To establish statistical relevance, at least three specimens for each test type were analyzed, with their data treated and validated as acceptable.

#### 2.3.1. mDCB Test

To test the mode I component, the mDCB, the previously described experimental setup (Figure 4) was used. The specimen was fixed on the loading hole of the mDCB lower bar (BC_1_) and loaded on the upper composed beam (BC_2_), i.e., the full ELS specimen. The final boundary condition was achieved by means of a clamp attached to a mobile base (BC_3_).

Following the removal of the ELS pre-crack blade, a folded Teflon^®^ sheet (Wilmington, DE, USA) with lubricant inside it was inserted in between the adherends of the ELS specimen. Both previous steps were performed to reduce friction at the ELS pre-crack region. The load–displacement curve was recorded, and then its data were analyzed using the CBBM.

##### Data Reduction—CBBM for mDCB Test

To correctly define the behavior of the novel mDCB specimen, a custom variation of CBBM was devised by Correia et al. [37]. As a result, the following expression for the mode I energy release rate of the mDCB specimen (Equation (1)) was obtained:

(1) $$G_{I}=\frac{P^{2}}{2\;b}\left(\left(\frac{12\;a_{eq\;I}^{2}}{E_{fI}\;b\;h_{I}^{3}}+\frac{6}{5\;G_{13}\;b\;h_{I}}\right)+\left(\frac{3\;a_{eq\;I}^{2}}{2\;E_{fII}\;b\;h_{II}^{3}}+\frac{3}{5\;G_{13}\;b\;h_{II}}\right)\right)$$

where *P* is the applied load, *b* the specimen width, *h*_I_ is the thickness of the mDCB substrate, *h*_II_ is the thickness of the ELS substrates, *G*_13_ is their shear modulus, *a*_eq I_ is the mDCB equivalent crack length, and (*E_f_*_I_; *E_f_*_II_) are the corrected flexural moduli for the mode I and mode II components of the formulation, respectively.

These last parameters (*E_f_*) are part of the CBBM formulation, as previously said in Section 1. Being corrected flexural moduli, they lose their meaning as material properties and become dependent on the specimen’s particularities. Since they consider the stress concentration and the substrates’ rotation near the crack tip, they are already defined for a standard DCB [9] and ELS [18].

As such, as described by Correia et al. [37], the mode I mDCB formulation was deduced considering the presence of both a DCB (lower beam) and an ELS (two upper beams), as clearly seen in Figure 2. This resulted in the presence of two corrected flexural moduli (*E_f_*_I_; *E_f_*_II_), one for each part of the formulation.

##### Compliance Ratio—Inclinometers

Additionally, having this mixed formulation resulted in the need to track the behavior of each mDCB arm individually. For this purpose, the rotation (*θ*) of each loading point was measured, and an initial compliance ratio, *r*, (Equation (2)) was calculated (see full CBBM formulation in [37]). Similarly to the initial compliance (*C*_0_) used in the CBBM, only the initial linear-elastic stage (*θ*^0^), pre-propagation, was relevant.

(2) $$r=\frac{\left|\theta_{I}^{0}\right|}{\left|\theta_{II}^{0}\right|+\left|\theta_{I}^{0}\right|}\;.$$

**Figure 5:**
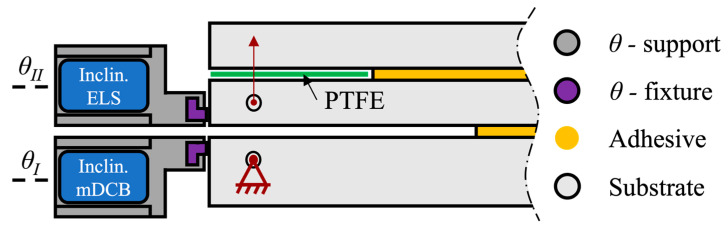
Inclinometer setup. *θ*-support in dark grey, and *θ*-fixture in purple.

As such, due to the need to determine experimentally the mDCB compliance ratio (*r*), custom fixtures were designed, as seen in Figure 5.

To hold the uniaxial TMS22E-PKH080 inclinometers (SICK—Waldkirch, Germany) in place, two supports (*θ*-support) were manufactured using a PRUSA (Prague, Czech Republic) 3D printer—model i3 MK3S—with a polylactic acid (PLA) polymer filament. And to fix the supports to each of the specimens, two slide-on fixtures (*θ*-fixture) were printed as well.

The *θ*-fixtures were glued in position with cyanoacrylate adhesive (Düsseldorf, Germany). The inclinometers connected to a data-acquisition system were positioned in the *θ*-supports and then connected to the specimen, as seen in Figure 5. For each test, the rotation values of each arm of the specimen were recorded, and the compliance ratios were calculated.

#### 2.3.2. ELS Test

The setup presented in Figure 4 was used for the mode II test, considering that the mode I test ended and lower substrate is fully debonded from the rest of the specimen. At this stage, the specimen is loaded on one side (BC_2_) while the opposite end is clamped vertically (BC_3_), being allowed to move within the horizontal degree of freedom.

Recall that, to avoid friction caused by the contact between the upper and lower adherends during testing, a green Teflon^®^ sheet coated with lubricant was previously inserted between the adherends in the pre-crack area (seen in Figure 2 and Figure 4).

The load and displacement for each test were recorded, and the fracture-energy release rate was calculated using the CBBM.

##### Data Reduction—CBBM for ELS Test

To attain the mode II fracture toughness recurring to CBBM, Equation (3) [18] was used:

(3) $$G_{II}=\frac{9P^{2}a_{eq\;II}^{2}}{4b^{2}E_{fII}h_{II}^{3}}F_{2}\;.$$

where *P* is the load applied, *b* is the specimen width, *h*_II_ is the thickness of the substrates, *a*_eq II_ the equivalent crack length, *E_f_*_II_ is the corrected flexural modulus, and *F*_2_ is a large-displacement correction factor.

## 3. Experimental Results and Discussion

**Figure 6:**
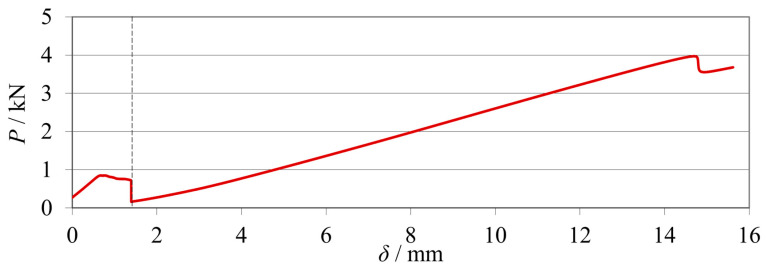
Representative *P*-*δ* curves of the sequential unified mode I (mDCB) and II (ELS) test.

As a preview of the final testing procedure, as previously mentioned, the mode I (mDCB) and II (ELS) fracture tests were merged in the same specimen. These were performed sequentially, and the load–displacement curves were registered by the machine, as seen in Figure 6.

The curves of each test can be easily distinguished by the failure of the mDCB specimen (near *δ* = 2 mm), and the mode II fracture test starts immediately after that. The resultant testing data would then be analyzed using a custom data-reduction code, which would split and treat each curve separately.

**Figure 7:**
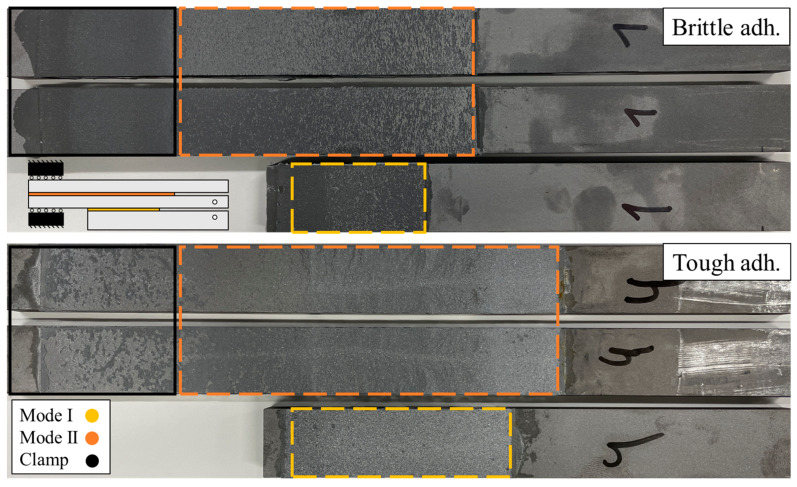
Representative fracture surfaces of the cohesive failure mode from unified mode I (mDCB) and II (ELS) tests of each adhesive used. Upper substrates are related with the brittle adhesive and lower substrates with the tough adhesive. For mode I, only one surface is shown since its counterpart is below one of the mode II substrates, as seen in the untested scheme of the unified specimen.

Post-testing, the fracture surfaces of each adhesive layer were analyzed, as seen in Figure 7. By searching for possible defects or adhesive failure areas that could negatively influence the tests, the results can be analyzed critically. As such, only results without defects and cohesive failure like the one from Figure 7 were considered for data treatment. Other than this, the actual crack length values can be measured from the failed specimens.

In this section, the experimental results are presented first and then discussed in each subsection. Two different conditions, a Balanced and an Unbalanced configuration, were tested experimentally. These nomenclatures mostly concern the behavior of the mDCB test, as previously detailed.

All experimental curves presented, both *P*-*δ* curves and R-curves, are representative of the data pool of each condition tested. However, at least three specimens for each test type were analyzed, with their data treated and validated as acceptable.

### 3.1. Balanced Specimen—r = 0.5

The Balanced configuration presents a (*h*_I_, *h*_II_) relation that results in a numerical mDCB compliance ratio (*r*, Equation (2)) of 0.5, which suggests the equal rotation of each specimen arm.

#### 3.1.1. Mode I—mDCB

**Figure 8:**
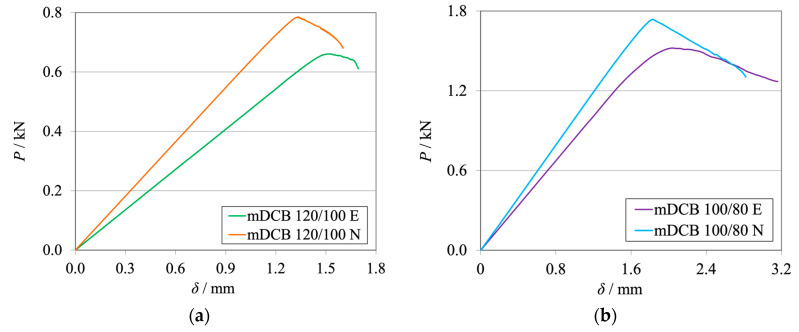
*P*-*δ* curves related to the Balanced mDCB specimen with *h*_II_ = 9 mm. Numerical (N) versus experimental (E) results. (**a**) Brittle adhesive; (**b**) Tough adhesive.

**Figure 9:**
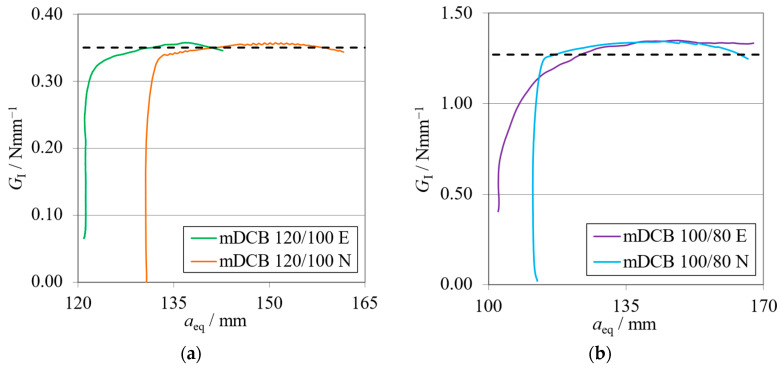
R-curves related to the Balanced mDCB specimen with *h*_II_ = 9 mm. Numerical (N) versus experimental (E) results. The black dashed line represents the mean *G*_IC_ value obtained with standard methods (Table 4). (**a**) Brittle adhesive; (**b**) Tough adhesive.

The *P*-*δ* curves and R-curves of the brittle and the tough adhesive for the Balanced specimen (*h*_II_ = 9 mm) are represented in Figure 8a and Figure 9a and Figure 8b and Figure 9b, respectively.

A summary of values obtained for all tests performed—mDCB for the unified specimen and DCB for the standard methods—is presented in Table 4. The relative differences (∆*G*) between the new data against the reference values (Table 1) is presented by means of a percentage.

**Figure 10:**
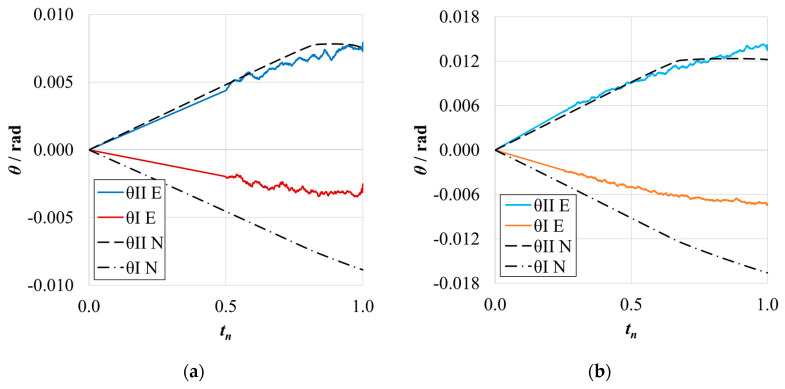
Rotation curves related to the Balanced mDCB specimen with *h*_II_ = 9 mm. Numerical (N) versus experimental (E) results. (**a**) Brittle adhesive; (**b**) Tough adhesive.

Representative curves of the arm rotations of the Balanced specimen are presented in Figure 10a for the brittle adhesive and Figure 10b for the tough adhesive.

The mean experimental values and respective standard deviations of each compliance ratio measured are presented in Table 5. Furthermore, the relative difference (∆*r*) between the experimental and numerical values is presented as well.

Looking at the machine’s results (Figure 8), it becomes evident that the stiffness of the numerical simulation is higher than the experimental results. This phenomenon is expected, since the experimental setup is more compliant than the rigid boundary conditions of the simulation. With lower stiffness, it is expected that the experimental crack initiates its propagation at both lower load and higher cross-head displacement. At the point where the adhesive reached the same stress state, which confers its characteristic fracture toughness (*G*_IC_), initiation begins.

As shown in the R-curves—Figure 9—the numerical and experimental mDCB specimen present highly similar behavior. When compared against their standard counterpart, the DCB test, which was presented by the black dashed lines, small relative errors were found. Table 4 shows variations smaller than 5% that fall within the result’s standard deviation range and are therefore considered negligible.

Having tracked the arm rotations (Figure 10) of each respective mDCB beam, their normalized behavior against the numerical results presented interesting findings. The upper arm—the ELS specimen—showed a good correlation against the numerical trend. However, the bottom arm—the mDCB arm—rotated less experimentally. This behavior was found for all tested specimens in all the studied mDCB configurations. As a result, the compliance ratios (Table 5) decreased by 38%, for the brittle adhesive, and 33%, for the tough adhesive.

Nonetheless, even though this change in behavior was observed, when looking at the results obtained for both adhesives, the actual *r* values felt by the specimen resulted in a proper characterization performance. This suggests that the compliance ratio depends not only on the specimen but also on the apparatus used. As such, this fact highlights the need to measure the arm rotations in order for the data-reduction scheme to account for the current conditions and properly characterize the adhesive.

This phenomenon might result from several facts: the lower rigidity of the apparatus, the effect of specimen self-weight, and others. This study proved that all these factors combined alter the load state of the specimen in relation to the numerical study but not its characterization capabilities.

#### 3.1.2. Mode II—ELS

**Figure 11:**
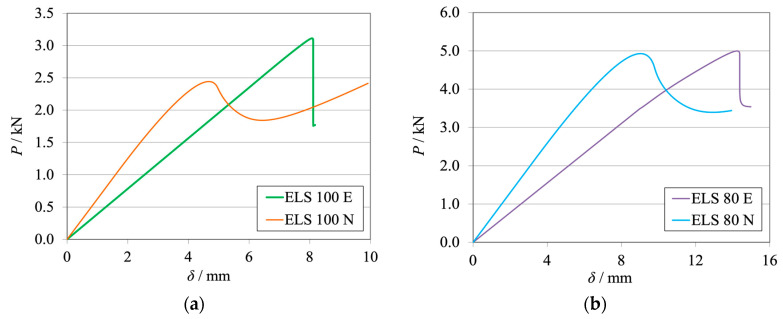
*P*-*δ* curves related to the ELS specimens with *h*_II_ = 9 mm. Numerical (N) versus experimental (E) results. (**a**) Brittle adhesive; (**b**) Tough adhesive.

**Figure 12:**
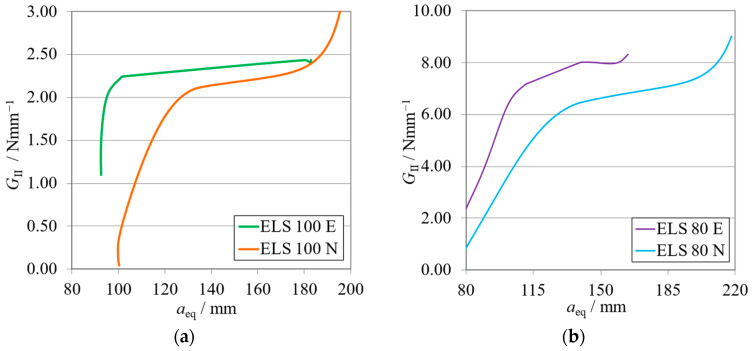
R-curves related to the ELS specimens with *h*_II_ = 9 mm. Numerical (N) versus experimental (E) results. (**a**) Brittle adhesive; (**b**) Tough adhesive.

The *P*-*δ* curves and R-curves for the ELS specimens with *h*_II_ = 9 mm are shown in Figure 11a and Figure 12a for the brittle adhesive and Figure 11b and Figure 12b for the tough adhesive.

The mean values and respective standard deviations of each characterization method used—ELS for the unified specimen and ENF for the standard methods (Table 1)—are presented in Table 6. Additionally, the relative difference (∆*G*) between these values is presented by means of a percentage.

As expected, it can be observed that numerical curves present higher stiffness than experimental ones. The proposed crack lengths of 100 mm (brittle) and 80 mm (tough) were chosen to reach a compromise between the need for *a*_0 I_ to be higher than *a*_0 II_ and the promotion of mode II stable crack propagation [37]. However, as seen in Figure 11a, this objective was not achieved for the brittle adhesive since all tests presented abrupt propagation stages (Figure 12a). Even though this behavior was found, the average value obtained for the brittle adhesive showed relative errors of 6%, falling within the standard deviation range of the reference ENF test.

For the tough adhesive, an important observation was made during the experimental tests since the substrates were bent near the clamp region, proving they were plastically deformed. This was also supported by the highly non-linear behavior present in Figure 11b, where a progressive reduction of stiffness is seen until propagation. Even when using high-strength steel (Table 2) of approximately 2180 MPa in ultimate strength, the 9 mm thick ELS specimens were deformed near the clamping tool. This phenomenon is associated with the increase in experimental compliance, which requires much larger deflections to achieve the same critical adhesive stress state. Meanwhile, the steel’s yield stress can be easily reached in the region near the clamp tool, deforming the specimen.

This plastic deformation increased the energy absorbed, leading to an overestimation of the mode II fracture toughness of this adhesive, as seen in Figure 12b. When looking solely at the mean values, this inaccuracy, resulting in errors of about 16% (Table 6), could be considered acceptable. However, there is no superposition between the ranges of the standard deviation of each method, discrediting the previous affirmation.

As stated above, the *G*_IIC_ of the tough adhesive was not correctly measured, since the substrates suffered plastic deformation. A solution to this issue lies in the use of thicker steel substrates also manufactured from high strength steel (Table 2); this solution can be proven in the study of the Unbalanced configuration, whose results are presented in the following section.

### 3.2. Unbalanced Specimen—r = 0.7

The Unbalanced configuration presents a (*h*_I_, *h*_II_) relation that results in a numerical mDCB compliance ratio (*r*, Equation (2)) of 0.7, which suggests uneven rotation of each specimen arm.

#### 3.2.1. Mode I—mDCB

**Figure 13:**
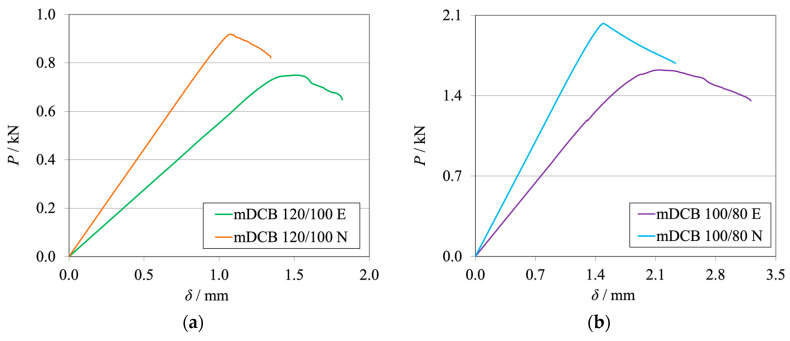
*P*-*δ* curves related to the Unbalanced mDCB specimen with *h*_II_ = 12.7 mm. Numerical (N) versus experimental (E) results. (**a**) Brittle adhesive; (**b**) Tough adhesive.

**Figure 14:**
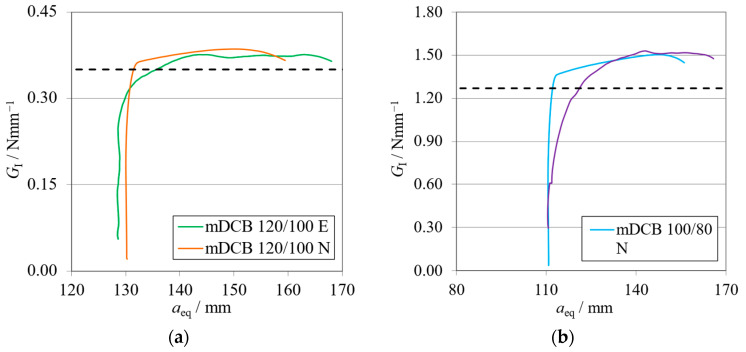
R-curves related to the Unbalanced mDCB specimen with *h*_II_ = 12.7 mm. Numerical (N) versus experimental (E) results. The black dashed line represents the mean *G*_IC_ value obtained with standard methods (Table 7). (**a**) Brittle adhesive; (**b**) Tough adhesive.

Figure 13a and Figure 14a for the brittle adhesive and Figure 13b and Figure 14b for the tough adhesive represent the *P*-*δ* curves and R-curves obtained for the Unbalanced specimens with *h*_II_ = 12.7 mm, respectively, for each adhesive.

The mean values and respective standard deviations of each characterization test performed—mDCB for the unified specimen and DCB for the standard methods (Table 1)—are presented in Table 7. The relative difference (∆*G*) between these values is presented as well.

**Figure 15:**
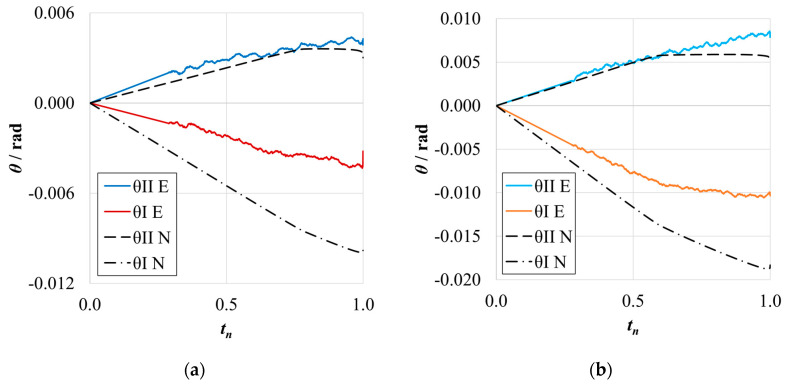
Rotation curves related to the Unbalanced mDCB specimen with *h*_II_ = 12.7 mm. Numerical (N) versus experimental (E) results. (**a**) Brittle adhesive; (**b**) Tough adhesive.

The rotation curves of the Unbalanced specimen are displayed in Figure 15a for the brittle adhesive and Figure 15b for the tough adhesive.

The mean experimental values and respective standard deviations of the measured compliance ratios are presented in Table 8. The relative difference (∆*r*) between the experimental and numerical values obtained for each condition are also presented.

Once more, the experimental results present lower stiffness and propagation at lower loads and higher displacements in relation to the numerical results, as seen in Figure 13.

Similarly to the Balanced configurations, both the R-curves—Figure 14—and the average *G*_IC_ experimental values presented a good resemblance to the respective numerically obtained data. However, when compared against their standard counterpart, higher relative errors were found, as presented in Table 7. Nonetheless, this was predicted in the numerical study, proving that the numerical behavior of the Unbalanced specimen is well defined. It is also relevant to say that the data-reduction scheme increases its overestimation of the higher fracture toughness of the adhesive.

The arm rotations (Figure 15) of each respective mDCB beam showed the same behavior as the Balanced ones, and once more, this trend was found for all tested specimens. The resulting compliance ratios, seen in Table 8, decreased by 38% for the brittle adhesive and 20% for the tough adhesive.

For this configuration, the change in *r* values in relation to the numerical results also benefited the characterization performance, resulting in a correct adhesive characterization when compared to the numerical predictions.

#### 3.2.2. Mode II—ELS

**Figure 16:**
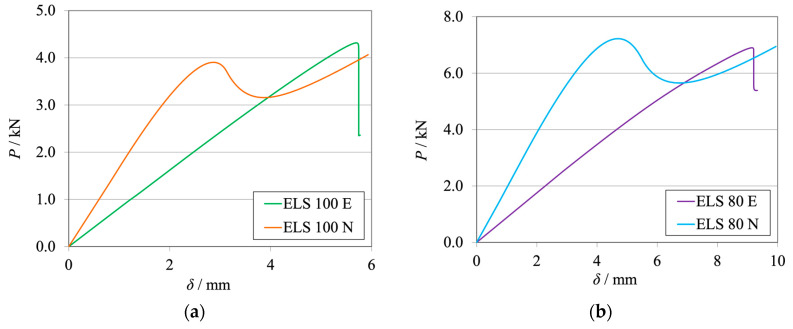
*P*-*δ* curves related to the ELS specimen with *h*_II_ = 12.7 mm. Numerical (N) versus experimental (E) results. (**a**) Brittle adhesive; (**b**) Tough adhesive.

**Figure 17:**
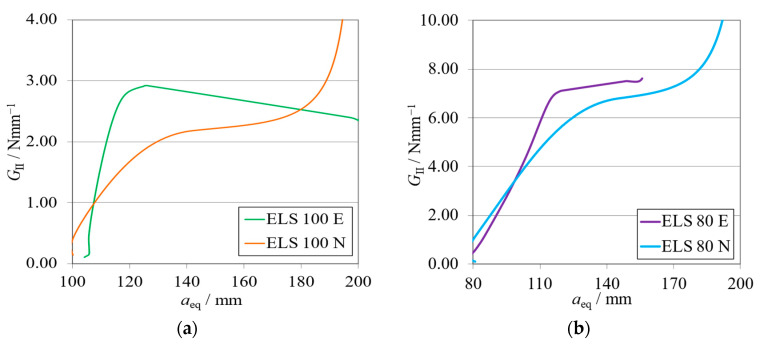
R-curves related to the ELS specimen with *h*_II_ = 12.7 mm. Numerical (N) versus experimental (E) results. (**a**) Brittle adhesive; (**b**) Tough adhesive.

The *P*-*δ* curves and R-curves of the ELS specimens with *h*_II_ = 12.7 mm are presented in Figure 16a and Figure 17a for the brittle adhesive and Figure 16b and Figure 17b for the tough adhesive.

A condensed presentation of values obtained for all tests performed—ELS for the unified specimen and ENF for the standard methods (Table 1)—is presented in Table 9. The relative differences (∆*G*) between the new data against the reference values are presented by means of a percentage.

Similarly to the previously presented ELS configuration, the numerical curves present higher stiffness, as is expected. The representative *P*-*δ* and R-curves for brittle adhesive, Figure 11a and Figure 17a, again depict unstable crack propagation around the fracture energy of the adhesive, *G*_IIC_. This unstable behavior required a high number of tests, resulting in a high standard deviation. Nonetheless, it was found that the mean value was satisfactory (Table 6) since the relative errors against the ENF test were simply 2%.

The tough adhesive specimens did not visibly present any plastic deformation, even when considering the smaller but still existing progressive reduction in stiffness, prior to propagation, presented in Figure 11b. This behavior can be attributed, in this case, to the FPZ plasticity present in more ductile adhesives. Since the specimen thickness was increased to 12.7 mm, and even though the high-strength steel now used had only 1740 MPa in ultimate strength, the extra thickness prevented plastic deformation. This was achieved by increasing the specimen’s stiffness and reducing its deflection by approximately 6 mm.

This change led to a proper measurement of the *G*_IIC_ of this adhesive, as seen in the representative R-curve in Figure 17b. As such, negligible differences between the ELS and ENF results were found for this configuration, as presented in Table 9.

From these results, it is possible to assess that the increase in thickness improved the prior problem of substrate yielding, but the propagation stability issue remains. As a solution, the mode II crack length could be further increased, but a compromise must be made in terms of the need for having enough useful mode I adhesive length while *a*_0 I_ is higher than *a*_0 II_. Therefore, the room for improvement in the ELS test, i.e., increasing *a*_0 II_ to promote mode II stable crack propagation, has a limit.

In the current state, the ELS test presents a limitation that results in the need for extensive mode II testing in order to have a sufficiently big data pool each time an adhesive is being characterized. This reduces the effects of having unpredictable/unstable crack propagation. As such, to prevent this problem, a conceptual change is preliminarily studied in the next section to assess the possibility of having a better mode II fracture specimen alternative while maintaining the good performance of the mode I test.

## 4. Conceptual Change—mDCB Plus I-ELS

Having proved both numerically and experimentally that the mDCB specimen presents a good characterization performance in relation to the standard DCB, we can state that the main issue regarding the combined specimen was the ELS specimen. The issues ranged from substrate yielding for thin specimens to crack propagation instabilities due to the high *a*_0_/*L* relations. Therefore, overall, the necessary compromise between the mDCB and ELS specimens does not make this concept fully viable.

**Figure 18:**
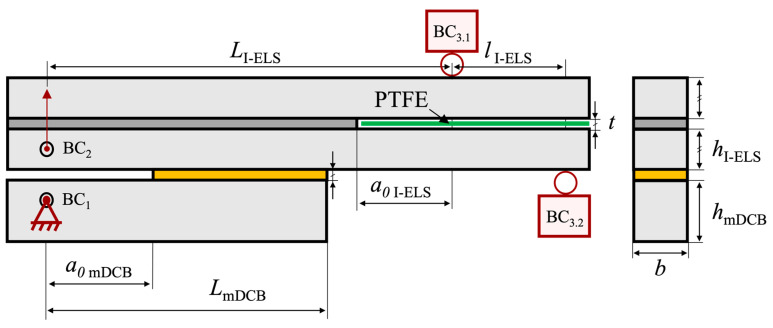
Schematic representation of the mDCB and I-ELS fracture specimen with its associated dimensions, testing procedure, and respective boundary conditions (BCs).

As such, an adaptation of the current concept was devised owing to the inverse end-loaded slip (I-ELS) specimen studied by Budzik and Jumel [26,27]. Being an inherently stable test and not presenting any need for a crack length compromise, all the ELS-related issues would be solved. Changing to this new concept resulted in the following combined specimen, presented in Figure 18.

Having a proof of concept for the I-ELS specimen [26], the only questions brought up by this adjustment are the modifications made to the mDCB specimen. As it no longer presents any mode II crack in the mode I CBBM formulations, it now must account for new boundary conditions introduced by the I-ELS rollers (BC_3.1_ and BC_3.2_). The distance between rollers is defined by the *l*_I-ELS_ parameter (Figure 18).

A preliminary numerical study was performed to seek answers to a few of these questions. A mDCB specimen was modeled under two conditions, considering the I-ELS roller boundary condition, active or inactive. Their data were analyzed through two methods, the mDCB CBBM considering *a*_0 II_ = 0 (Equation (1)) and the standard DCB CBBM [9]. The numerical procedures used were similar to the ones presented by Correia et al. [37], simply adapting the simulations to the I-ELS concept.

The mDCB initial crack length was set at 45 mm. All other dimensions were considered as not relevant for this work and are to be the subject of future work.

### Mode I Component—mDCB Plus I-ELS

**Figure 19:**
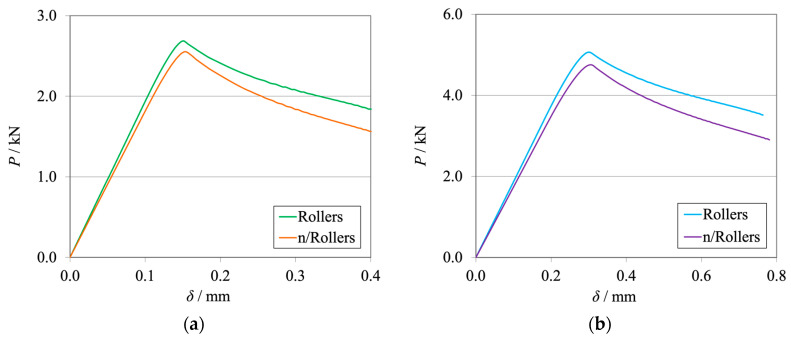
*P*-*δ* curves related to the mDCB specimen of the novel concept integrating the I-ELS mode II component. (**a**) Brittle adhesive; (**b**) Tough adhesive.

The *P*-*δ* curves of each condition tested are presented in Figure 19a for the brittle adhesive and Figure 19b for the tough adhesive.

**Figure 20:**
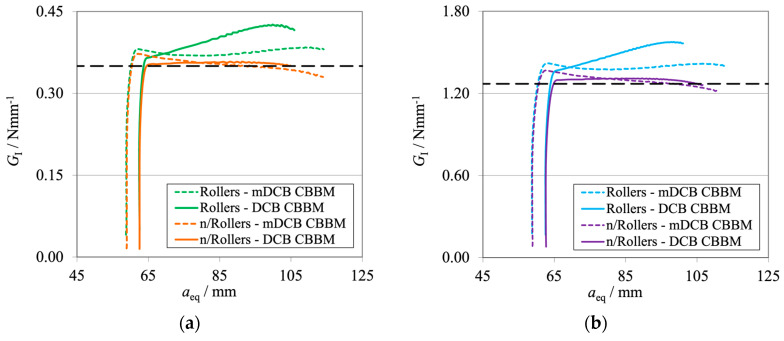
R-curves related to the mDCB specimen of the novel concept integrating the I-ELS mode II component. The black dashed line represents the input *G*_IC_ value obtained with experimental tests (Table 1). (**a**) Brittle adhesive; (**b**) Tough adhesive.

The corresponding R-curves computed using the mDCB and DCB CBBM are presented in Figure 20, for both active (in green or blue) and inactive (in orange or purple) rollers.

For both adhesives, the load supported by the specimen and its stiffness are higher for the conditions where the rollers are active (Figure 19). This is due to the fact that part of the load is supported by the rollers since they are restricting the free movement of the specimen. Then, lowering the severity of the damage in the mDCB crack results in the need for higher load values to achieve the same critical stress state.

Regarding the R-curves (Figure 20), it is possible to see that the trends are the same independently of the adhesive; therefore, the conclusions are the same for both adhesives used.

Considering the I-ELS rollers as an active boundary condition and using the mDCB data reduction scheme (green and blue dashed lines) resulted in a slight overestimation of the *G*_IC_ values. The curves also presented a small oscillation or waviness around their average *G*_IC_ value. When considering the DCB CBBM as a reduction procedure (green and blue lines), both curves presented a ramp during propagation, without a *G*_IC_ plateau being attained.

When the I-ELS rollers as a boundary condition of the mDCB test were removed, all curves presented a correct average value of *G*_IC_. Overall, the standard DCB data reduction scheme (orange and purple lines) presented a better characterization behavior since the R-curve was both precise and stable in relation to the input property values.

However, when applying the mDCB data reduction scheme, the curves did not present a proper *G*_IC_ plateau. Crack propagation began at a higher value of *G*_I_ and then decreased slowly until the correct value was obtained, but it never presented a well-defined plateau. This peak phenomenon was also seen in the previous numerical study when considering small crack lengths [28]. Nonetheless, the simpler DCB CBBM data-reduction scheme proved to be a much better option. This conclusion is supported by the resultant R-curves and does not require the calculation of a compliance ratio.

## 5. Conclusions

In this experimental study, the use of combined mDCB and ELS tests was experimentally validated, using two different structural adhesives, against the isolated methods commonly used, the DCB test and the ENF test.

Two configurations, referred to as Balanced and Unbalanced, were analyzed to assess certain practical issues related with the FPZ, substrate yielding and interactions between crack lengths.

From this study, the following conclusions were made:

- Both the Balanced and Unbalanced mDCB specimens presented a favorable correlation between the numerical and experimental results.
- When compared against standard methods, the Balanced mDCB showed good equivalence; however, some divergences appeared for the Unbalanced one, which were expected from the numerical study.
- For mode II characterization, in the Balanced configuration, the use of the recommended crack length did not prove effective enough in fighting the propagation instability of the brittle adhesive.
- The use of slim 9 mm ELS substrates resulted in the presence of plastic deformation for the tough adhesive.
- The use of thicker ELS substrates (Unbalanced specimen) improved the plasticity concerns found earlier, but the stability issues remained for the brittle adhesive.
- The use of the I-ELS test, instead of the ELS, has shown promising results regarding the mode I combined test due to the simpler specimen design considerations and data-reduction methods necessary.

Overall, the numerical performance of the mode I mDCB test was successfully experimentally validated, showing that the specimen and data-reduction methods are robust enough to adapt to real conditions while extracting the correct adhesive properties.

As for the mode II test, the compromise made to ensure the combined character of the mDCB-plus-ELS test introduced issues related with substrate plasticity and crack propagation instability. Nonetheless, the preliminary results on the use of the I-ELS to improve the current concept have shown to be good prospects for the future of this project.

## Figures and Tables

**Table 1:** Fracture properties obtained experimentally through standardized testing.

Property	Brittle Adhesive ^1^	Tough Adhesive ^2^
*G*_IC_/Nmm^−1^	0.35 ± 0.08	1.27 ± 0.05
*G*_IIC_/Nmm^−1^	2.40 ± 0.38	7.22 ± 0.33

Obtained experimentally in ^1^ [37], ^2^ [39].

**Table 2:** Mechanical properties of the substrates used. Steel hardness (*H*) through the Rockwell test, the ultimate tensile strength *(σ_r_*), and the thickness of the substrate (*h*).

Property	DIN 40CrMnMo7	DIN X155CrVMo12-1
*H*/HRC	51	64
*σ_r_*/MPa	≈1740	>2180
*h*/mm	12.7	9

**Table 3:** Geometrical parameters (see Figure 2) of the unified specimen [37]. Dimensions in millimeters.

Config.	*L*_II_	*L*_I_	*a*_0 II_	*a*_0 I_	*h*_II_	*h*_I_	*L*_Clamp_	*b*	*t*
Balanced	290	190	100 ^1^/80 ^2^	120 ^1^/100 ^2^	9	12.7	70	25	0.2
Unbalanced					12.7				

Related to the ^1^ Brittle adh. (Unchanged from [37]), ^2^ Tough adh. (Altered from [37]).

**Table 4:** Mode I critical energy release rate, *G*_IC_, for Balanced mDCB specimen with *h*_II_ = 9 mm versus the standard specimen, the DCB test.

Adhesive	*G*_IC mDCB_/Nmm^−1^	*G*_IC DCB_/Nmm^−1^	Δ*G/*%
Brittle	0.34 ± 0.02	0.35 ± 0.08	−3
Tough	1.31 ± 0.07	1.27 ± 0.05	+3

**Table 5:** Compliance ratios, *r*, for the Balanced mDCB specimen with *h*_II_ = 9 mm. Numerical (N) versus experimental (E) results.

Adhesive	*r*_N_	*r*_E_	∆*r*/%
Brittle	0.50	0.30 ± 0.07	−38
Tough	0.50	0.33 ± 0.04	−33

**Table 6:** Mode II critical energy release rate, *G*_IIC_, for the ELS specimen with *h*_II_ = 9 mm versus the standard specimen, the ENF test.

Adhesive	*G*_IIC ELS_/Nmm^−1^	*G*_IIC ENF_/Nmm^−1^	Δ*G/*%
Brittle	2.55 ± 0.32	2.40 ± 0.38	+6
Tough	8.42 ± 0.79	7.22 ± 0.33	+17

**Table 7:** Mode I critical energy release rate, *G*_IC_, for the Unbalanced mDCB specimen with *h*_II_ = 12.7 mm versus the standard specimen, the DCB test.

Adhesive	*G*_IC mDCB_/Nmm^−1^	*G*_IC DCB_/Nmm^−1^	Δ*G/*%
Brittle	0.34 ± 0.02	0.35 ± 0.08	−3
Tough	1.31 ± 0.07	1.27 ± 0.05	+3

**Table 8:** Compliance ratios, *r*, for the Unbalanced mDCB specimen with *h*_II_ = 12.7 mm. Numerical (N) versus experimental (E) results.

Adhesive	*r*_N_	*r*_E_	Δ*r*/%
Brittle	0.70	0.43 ± 0.01	−38
Tough	0.70	0.56 ± 0.03	−20

**Table 9:** Mode II critical energy release rate, *G*_IIC_, for the ELS specimen with *h*_II_ = 12.7 mm versus the standard specimen, the ENF test.

Adhesive	*G*_IIC ELS_/Nmm^−1^	*G*_IIC ENF_/Nmm^−1^	Δ*G*/%
Brittle	2.44 ± 0.54	2.40 ± 0.38	+2
Tough	7.26 ± 0.41	7.22 ± 0.33	+1

## Data Availability

Data are contained within the article.
